# 1-Allyl-4-(1,3-benzothia­zol-2-yl)-5-methyl-2-phenyl-1*H*-pyrazol-3(2*H*)-one

**DOI:** 10.1107/S1600536810040316

**Published:** 2010-10-20

**Authors:** Imane Chakib, Abdelfettah Zerzouf, Hafid Zouihri, El Mokhtar Essassi, Seik Weng Ng

**Affiliations:** a Laboratoire de Chimie Organique Hétérocyclique, Pôle de Compétences Pharmacochimie, Université Mohammed V-Agdal, BP 1014 Avenue Ibn Batout, Rabat, Morocco; b Department of Chemistry, University of Malaya, 50603 Kuala Lumpur, Malaysia

## Abstract

The title compound, C_20_H_17_N_3_OS, is a 1*H*-pyrazol-3(2*H*)-one having aromatic 4-(1,3-benzothia­zol-2-yl)- and 2-phenyl substituents. The five-membered ring and fused ring system are planar, the r.m.s. deviations being 0.021 and 0.005 Å, respectively. The five-membered ring is aligned at 7.9 (2)° with respect to the fused-ring system. The allyl and phenyl parts of the mol­ecule are both disordered over two positions in a 1:1 ratio. Weak inter­molecular C—H⋯O hydrogen bonding is present in the crystal structure.

## Related literature

For the structure of a related compound (*E*)-4-(2,3-dihydro-1,3-benzothia­zol-2-yl­idene)-3-methyl-1-phenyl-1*H*-pyrazol-5(4*H*)-one, see: Chakibe *et al.* (2010 ▶).
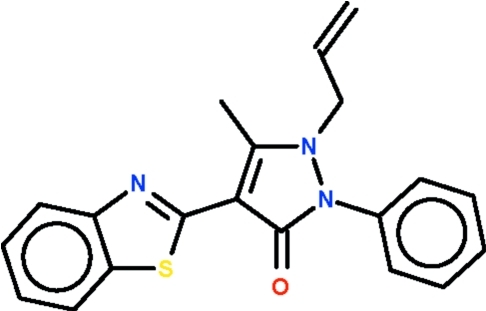


## Experimental

### 

#### Crystal data

C_20_H_17_N_3_OS
*M* *_r_* = 347.43
Orthorhombic, 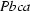

*a* = 17.8734 (5) Å
*b* = 10.4297 (2) Å
*c* = 18.9578 (4) Å
*V* = 3534.00 (14) Å^3^
*Z* = 8
Mo *K*α radiation
μ = 0.20 mm^−1^
*T* = 293 K
0.30 × 0.30 × 0.25 mm

#### Data collection

Bruker X8 APEXII diffractometer
Absorption correction: multi-scan (*SADABS*; Sheldrick, 1996 ▶) *T*_min_ = 0.944, *T*_max_ = 0.953
18349 measured reflections
3678 independent reflections
2341 reflections with *I* > 2σ(*I*)
*R*_int_ = 0.032

#### Refinement

*R*[*F* ^2^ > 2σ(*F* ^2^)] = 0.050
*wR*(*F* ^2^) = 0.172
*S* = 1.00
3678 reflections
227 parameters
17 restraints
H-atom parameters constrained
Δρ_max_ = 0.32 e Å^−3^
Δρ_min_ = −0.25 e Å^−3^

### 

Data collection: *APEX2* (Bruker, 2008 ▶); cell refinement: *SAINT* (Bruker, 2008 ▶); data reduction: *SAINT*; program(s) used to solve structure: *SHELXS97* (Sheldrick, 2008 ▶); program(s) used to refine structure: *SHELXL97* (Sheldrick, 2008 ▶); molecular graphics: *X-SEED* (Barbour, 2001 ▶); software used to prepare material for publication: *publCIF* (Westrip, 2010 ▶).

## Supplementary Material

Crystal structure: contains datablocks global, I. DOI: 10.1107/S1600536810040316/xu5047sup1.cif

Structure factors: contains datablocks I. DOI: 10.1107/S1600536810040316/xu5047Isup2.hkl

Additional supplementary materials:  crystallographic information; 3D view; checkCIF report

## Figures and Tables

**Table 1:** Hydrogen-bond geometry (Å, °)

*D*—H⋯*A*	*D*—H	H⋯*A*	*D*⋯*A*	*D*—H⋯*A*
C2—H2⋯O1^i^	0.93	2.59	3.318 (3)	135
C12—H12*A*⋯O1^ii^	0.97	2.51	3.404 (4)	152
C12—H12*C*⋯O1^ii^	0.97	2.48	3.404 (4)	159

Symmetry codes: (i) 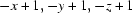; (ii) 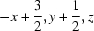.

**Fig. 1.**
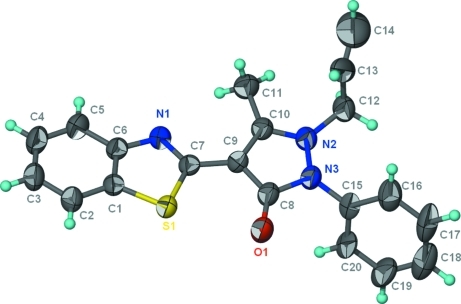
Thermal ellipsoid plot (Barbour, 2001) of C20H17N3OS at the 50% probability level; hydrogen atoms are drawn as arbitrary radius. The disorder is not shown

## supplementary crystallographic information

### Comment

(*E*)-4-(2,3-Dihydro-1,3-benzothiazol-2-ylidene)-3-methyl-1-phenyl-1*H*-pyrazol-5(4*H*)-one is an amine that can under a nucleophilic substitution with organo bromides to form 2-substituted derivatives if tetra-*n*-butyl ammonium bromide is used as catalyst. In this study, the compound is reacted with allyl bromide to yield the title compound (Scheme I, Fig. 1).

### Experimental

To a solution of (*E*)-4-(2,3-dihydro-1,3-benzothiazol-2-ylidene)-3-methyl-1-phenyl-1*H*-pyrazol-5(4*H*)-one (1 g, 3.25 mmol) in DMF (50 ml), was added sodium carbonate (2.5 g, 23 mmol), tetra-*n*-butyl ammonium bromide (0.15 g, 1 mmol) and allyl bromide (5.6 g, 46 mmol). The mixture was stirred for 24 h at room temperature. The solid material was removed by filtration and the solution was evaporated under reduced. The residue was washed with dichloromethane and hexane, and the recrystallized from ethanol to afford the title compound as colorless crystals.

### Refinement

Carbon-bound H-atoms were placed in calculated positions (C—H 0.93–0.97 Å) and were included in the refinement in the riding model approximation, with *U*(H) set to 1.2–1.5*U*_eq_(C).

The allyl and phenyl units are disordered over two positions; the disorder could be refined, and was assumed to be a 1:1 type of disorder. For the allyl unit, the single-bond distances were restrained to 1.50±0.01 Å and the double-bond distances to 1.35±0.01 Å; the anisotropic temperature factors were restrained to be nearly isotropic. The phenyl rings were refined as rigid hexagons of 1.39 Å sides; the N–C_phenyl_ pair of distances were restrained to within 0.01 Å of each other. Additionally, the temperature factors of the primed atoms were restrained to those of the unprimed ones.

### Crystal data

**Table:**

C_20_H_17_N_3_OS	*F*(000) = 1456
*M**_r_* = 347.43	*D*_x_ = 1.306 Mg m^−^^3^
Orthorhombic, *P**b**c**a*	Mo *K*α radiation, λ = 0.71073 Å
Hall symbol: -P 2ac 2ab	Cell parameters from 3745 reflections
*a* = 17.8734 (5) Å	θ = 2.3–22.5°
*b* = 10.4297 (2) Å	µ = 0.20 mm^−^^1^
*c* = 18.9578 (4) Å	*T* = 293 K
*V* = 3534.00 (14) Å^3^	Prism, colorless
*Z* = 8	0.30 × 0.30 × 0.25 mm

### Data collection

**Table:**

Bruker X8 APEXII diffractometer	3678 independent reflections
Radiation source: fine-focus sealed tube	2341 reflections with *I* > 2σ(*I*)
graphite	*R*_int_ = 0.032
φ and ω scans	θ_max_ = 26.6°, θ_min_ = 2.4°
Absorption correction: multi-scan (*SADABS*; Sheldrick, 1996)	*h* = −22→21
*T*_min_ = 0.944, *T*_max_ = 0.953	*k* = −13→12
18349 measured reflections	*l* = −23→23

### Refinement

**Table:**

Refinement on *F*^2^	Primary atom site location: structure-invariant direct methods
Least-squares matrix: full	Secondary atom site location: difference Fourier map
*R*[*F*^2^ > 2σ(*F*^2^)] = 0.050	Hydrogen site location: inferred from neighbouring sites
*wR*(*F*^2^) = 0.172	H-atom parameters constrained
*S* = 1.00	*w* = 1/[σ^2^(*F*_o_^2^) + (0.096*P*)^2^ + 0.8646*P*] where *P* = (*F*_o_^2^ + 2*F*_c_^2^)/3
3678 reflections	(Δ/σ)_max_ = 0.001
227 parameters	Δρ_max_ = 0.32 e Å^−^^3^
17 restraints	Δρ_min_ = −0.25 e Å^−^^3^

### Fractional atomic coordinates and isotropic or equivalent isotropic displacement parameters (Å^2^)

**Table:**

	*x*	*y*	*z*	*U*_iso_*/*U*_eq_	Occ. (<1)
S1	0.54022 (4)	0.59623 (7)	0.57529 (3)	0.0590 (2)	
O1	0.63189 (12)	0.5096 (2)	0.45761 (10)	0.0808 (6)	
N1	0.61613 (12)	0.7853 (2)	0.62953 (11)	0.0590 (6)	
N2	0.79529 (13)	0.6762 (2)	0.48741 (11)	0.0593 (6)	
N3	0.75432 (13)	0.5809 (2)	0.45335 (11)	0.0627 (6)	
C1	0.50073 (15)	0.6812 (2)	0.64448 (12)	0.0531 (6)	
C2	0.43115 (17)	0.6640 (3)	0.67650 (14)	0.0669 (7)	
H2	0.3989	0.5995	0.6617	0.080*	
C3	0.41164 (19)	0.7448 (3)	0.73035 (15)	0.0762 (9)	
H3	0.3654	0.7349	0.7522	0.091*	
C4	0.4595 (2)	0.8412 (3)	0.75287 (16)	0.0798 (9)	
H4	0.4454	0.8943	0.7900	0.096*	
C5	0.52717 (19)	0.8589 (3)	0.72110 (15)	0.0743 (8)	
H5	0.5587	0.9242	0.7362	0.089*	
C6	0.54904 (16)	0.7785 (2)	0.66569 (12)	0.0566 (6)	
C7	0.61898 (14)	0.6956 (2)	0.58142 (11)	0.0495 (6)	
C8	0.68208 (17)	0.5800 (3)	0.48056 (13)	0.0603 (7)	
C9	0.68293 (15)	0.6721 (2)	0.53608 (12)	0.0518 (6)	
C10	0.75276 (15)	0.7255 (2)	0.53898 (12)	0.0548 (6)	
C11	0.78327 (19)	0.8222 (3)	0.58934 (16)	0.0760 (8)	
H11A	0.8269	0.8617	0.5693	0.114*	
H11B	0.7965	0.7806	0.6327	0.114*	
H11C	0.7461	0.8865	0.5984	0.114*	
C12	0.87637 (17)	0.6862 (3)	0.48056 (16)	0.0746 (8)	
H12A	0.8914	0.7755	0.4806	0.090*	0.50
H12B	0.8926	0.6475	0.4366	0.090*	0.50
H12C	0.8825	0.7782	0.4852	0.090*	0.50
H12D	0.8812	0.6708	0.4303	0.090*	0.50
C13	0.9121 (11)	0.6151 (15)	0.5440 (7)	0.101 (3)	0.50
H13	0.8915	0.5398	0.5616	0.121*	0.50
C14	0.9722 (11)	0.6652 (18)	0.5718 (9)	0.139 (4)	0.50
H14A	0.9920	0.7407	0.5535	0.166*	0.50
H14B	0.9953	0.6254	0.6099	0.166*	0.50
C13'	0.9175 (11)	0.5938 (15)	0.5294 (7)	0.101 (3)	0.50
H13'	0.9148	0.5076	0.5171	0.121*	0.50
C14'	0.9555 (11)	0.6196 (18)	0.5854 (8)	0.139 (4)	0.50
H14C	0.9603	0.7041	0.6004	0.166*	0.50
H14D	0.9781	0.5538	0.6107	0.166*	0.50
C15	0.7691 (10)	0.5140 (10)	0.3885 (4)	0.0510 (18)	0.50
C16	0.8277 (10)	0.4304 (14)	0.3741 (5)	0.0863 (18)	0.50
H16	0.8561	0.3969	0.4108	0.104*	0.50
C17	0.8437 (10)	0.3967 (13)	0.3048 (5)	0.105 (3)	0.50
H17	0.8829	0.3407	0.2951	0.126*	0.50
C18	0.8012 (11)	0.4466 (10)	0.2499 (4)	0.110 (4)	0.50
H18	0.8119	0.4241	0.2035	0.132*	0.50
C19	0.7426 (10)	0.5303 (12)	0.2643 (6)	0.102 (3)	0.50
H19	0.7142	0.5637	0.2275	0.123*	0.50
C20	0.7265 (9)	0.5640 (12)	0.3336 (7)	0.0714 (19)	0.50
H20	0.6874	0.6199	0.3432	0.086*	0.50
C15'	0.7791 (10)	0.5424 (10)	0.3848 (5)	0.0510 (18)	0.50
C16'	0.8326 (10)	0.4458 (14)	0.3884 (4)	0.0863 (18)	0.50
H16'	0.8571	0.4294	0.4308	0.104*	0.50
C17'	0.8494 (11)	0.3738 (12)	0.3288 (5)	0.105 (3)	0.50
H17'	0.8852	0.3092	0.3312	0.126*	0.50
C18'	0.8127 (11)	0.3984 (9)	0.2656 (4)	0.110 (4)	0.50
H18'	0.8240	0.3502	0.2257	0.132*	0.50
C19'	0.7593 (10)	0.4949 (13)	0.2620 (5)	0.102 (3)	0.50
H19'	0.7347	0.5113	0.2197	0.123*	0.50
C20'	0.7425 (9)	0.5669 (11)	0.3216 (7)	0.0714 (19)	0.50
H20'	0.7067	0.6315	0.3192	0.086*	0.50

### Atomic displacement parameters (Å^2^)

**Table:**

	*U*^11^	*U*^22^	*U*^33^	*U*^12^	*U*^13^	*U*^23^
S1	0.0542 (4)	0.0698 (4)	0.0528 (4)	−0.0033 (3)	0.0062 (3)	−0.0106 (3)
O1	0.0647 (13)	0.1020 (15)	0.0756 (13)	−0.0228 (12)	0.0166 (10)	−0.0337 (12)
N1	0.0559 (14)	0.0630 (12)	0.0580 (12)	0.0042 (10)	0.0020 (10)	−0.0066 (10)
N2	0.0529 (14)	0.0655 (12)	0.0594 (12)	−0.0072 (11)	0.0080 (10)	−0.0040 (10)
N3	0.0568 (14)	0.0740 (13)	0.0573 (12)	−0.0097 (11)	0.0118 (11)	−0.0143 (10)
C1	0.0521 (16)	0.0634 (14)	0.0438 (12)	0.0102 (12)	−0.0007 (11)	0.0048 (10)
C2	0.0615 (18)	0.0812 (18)	0.0581 (15)	0.0026 (15)	0.0106 (13)	0.0007 (13)
C3	0.067 (2)	0.100 (2)	0.0611 (16)	0.0151 (18)	0.0163 (15)	0.0006 (15)
C4	0.084 (2)	0.090 (2)	0.0657 (17)	0.0216 (19)	0.0131 (17)	−0.0169 (16)
C5	0.075 (2)	0.0803 (18)	0.0677 (17)	0.0077 (16)	0.0047 (16)	−0.0213 (15)
C6	0.0589 (17)	0.0610 (14)	0.0500 (13)	0.0111 (13)	−0.0020 (12)	−0.0043 (11)
C7	0.0514 (15)	0.0534 (12)	0.0438 (11)	0.0041 (11)	−0.0018 (10)	0.0018 (10)
C8	0.0599 (17)	0.0689 (15)	0.0521 (13)	−0.0055 (14)	0.0088 (13)	−0.0060 (12)
C9	0.0538 (16)	0.0566 (13)	0.0450 (12)	0.0004 (12)	0.0022 (11)	−0.0006 (10)
C10	0.0558 (16)	0.0580 (13)	0.0507 (13)	−0.0014 (12)	0.0024 (12)	−0.0003 (11)
C11	0.070 (2)	0.0852 (19)	0.0725 (17)	−0.0151 (16)	0.0033 (15)	−0.0177 (15)
C12	0.0580 (19)	0.090 (2)	0.0763 (19)	−0.0105 (16)	0.0148 (15)	−0.0134 (16)
C13	0.072 (3)	0.124 (5)	0.108 (6)	0.039 (3)	−0.021 (4)	−0.065 (4)
C14	0.136 (8)	0.165 (9)	0.115 (6)	0.033 (7)	0.001 (5)	0.023 (6)
C13'	0.072 (3)	0.124 (5)	0.108 (6)	0.039 (3)	−0.021 (4)	−0.065 (4)
C14'	0.136 (8)	0.165 (9)	0.115 (6)	0.033 (7)	0.001 (5)	0.023 (6)
C15	0.059 (4)	0.031 (4)	0.0631 (17)	−0.024 (4)	0.0240 (16)	−0.001 (2)
C16	0.087 (3)	0.067 (3)	0.105 (4)	0.001 (3)	0.026 (4)	−0.022 (3)
C17	0.122 (5)	0.085 (5)	0.108 (8)	0.000 (4)	0.058 (7)	−0.028 (5)
C18	0.133 (8)	0.098 (8)	0.098 (5)	−0.045 (8)	0.057 (6)	−0.038 (5)
C19	0.100 (7)	0.149 (8)	0.0579 (19)	−0.034 (6)	0.029 (3)	−0.017 (3)
C20	0.069 (6)	0.102 (2)	0.043 (4)	−0.020 (3)	0.028 (3)	0.007 (2)
C15'	0.059 (4)	0.031 (4)	0.0631 (17)	−0.024 (4)	0.0240 (16)	−0.001 (2)
C16'	0.087 (3)	0.067 (3)	0.105 (4)	0.001 (3)	0.026 (4)	−0.022 (3)
C17'	0.122 (5)	0.085 (5)	0.108 (8)	0.000 (4)	0.058 (7)	−0.028 (5)
C18'	0.133 (8)	0.098 (8)	0.098 (5)	−0.045 (8)	0.057 (6)	−0.038 (5)
C19'	0.100 (7)	0.149 (8)	0.0579 (19)	−0.034 (6)	0.029 (3)	−0.017 (3)
C20'	0.069 (6)	0.102 (2)	0.043 (4)	−0.020 (3)	0.028 (3)	0.007 (2)

### Geometric parameters (Å, °)

**Table:**

S1—C1	1.733 (2)	C12—H12C	0.9701
S1—C7	1.752 (3)	C12—H12D	0.9701
O1—C8	1.238 (3)	C13—C14	1.305 (10)
N1—C7	1.307 (3)	C13—H13	0.9300
N1—C6	1.383 (3)	C14—H14A	0.9300
N2—C10	1.341 (3)	C14—H14B	0.9300
N2—N3	1.393 (3)	C13'—C14'	1.289 (9)
N2—C12	1.459 (3)	C13'—H13'	0.9300
N3—C8	1.391 (4)	C14'—H14C	0.9300
N3—C15'	1.430 (6)	C14'—H14D	0.9300
N3—C15	1.438 (6)	C15—C16	1.3900
C1—C6	1.392 (4)	C15—C20	1.3900
C1—C2	1.395 (4)	C16—C17	1.3900
C2—C3	1.369 (4)	C16—H16	0.9300
C2—H2	0.9300	C17—C18	1.3900
C3—C4	1.388 (5)	C17—H17	0.9300
C3—H3	0.9300	C18—C19	1.3900
C4—C5	1.363 (4)	C18—H18	0.9300
C4—H4	0.9300	C19—C20	1.3900
C5—C6	1.399 (4)	C19—H19	0.9300
C5—H5	0.9300	C20—H20	0.9300
C7—C9	1.451 (3)	C15'—C16'	1.3900
C8—C9	1.425 (3)	C15'—C20'	1.3900
C9—C10	1.368 (4)	C16'—C17'	1.3900
C10—C11	1.492 (4)	C16'—H16'	0.9300
C11—H11A	0.9600	C17'—C18'	1.3900
C11—H11B	0.9600	C17'—H17'	0.9300
C11—H11C	0.9600	C18'—C19'	1.3900
C12—C13'	1.525 (9)	C18'—H18'	0.9300
C12—C13	1.551 (8)	C19'—C20'	1.3900
C12—H12A	0.9700	C19'—H19'	0.9300
C12—H12B	0.9700	C20'—H20'	0.9300
			
C1—S1—C7	88.53 (12)	H12A—C12—H12B	108.5
C7—N1—C6	110.1 (2)	C13'—C12—H12C	121.0
C10—N2—N3	108.3 (2)	C13—C12—H12C	110.8
C10—N2—C12	126.9 (2)	C13'—C12—H12D	116.7
N3—N2—C12	122.1 (2)	H12C—C12—H12D	104.1
C8—N3—N2	108.7 (2)	C14—C13—C12	117.5 (15)
C8—N3—C15'	128.5 (7)	C14—C13—H13	121.3
N2—N3—C15'	117.3 (6)	C12—C13—H13	121.3
C8—N3—C15	118.9 (7)	C13—C14—H14A	120.0
N2—N3—C15	130.3 (6)	C13—C14—H14B	120.0
C6—C1—C2	121.4 (2)	H14A—C14—H14B	120.0
C6—C1—S1	109.81 (19)	C14'—C13'—C12	128.4 (15)
C2—C1—S1	128.8 (2)	C14'—C13'—H13'	115.8
C3—C2—C1	118.2 (3)	C12—C13'—H13'	115.8
C3—C2—H2	120.9	C13'—C14'—H14C	120.0
C1—C2—H2	120.9	C13'—C14'—H14D	120.0
C2—C3—C4	121.2 (3)	H14C—C14'—H14D	120.0
C2—C3—H3	119.4	C16—C15—C20	120.0
C4—C3—H3	119.4	C16—C15—N3	127.6 (11)
C5—C4—C3	120.6 (3)	C20—C15—N3	111.0 (10)
C5—C4—H4	119.7	C15—C16—C17	120.0
C3—C4—H4	119.7	C15—C16—H16	120.0
C4—C5—C6	119.9 (3)	C17—C16—H16	120.0
C4—C5—H5	120.0	C18—C17—C16	120.0
C6—C5—H5	120.0	C18—C17—H17	120.0
N1—C6—C1	115.6 (2)	C16—C17—H17	120.0
N1—C6—C5	125.7 (3)	C17—C18—C19	120.0
C1—C6—C5	118.7 (3)	C17—C18—H18	120.0
N1—C7—C9	124.4 (2)	C19—C18—H18	120.0
N1—C7—S1	115.99 (19)	C20—C19—C18	120.0
C9—C7—S1	119.57 (17)	C20—C19—H19	120.0
O1—C8—N3	123.1 (2)	C18—C19—H19	120.0
O1—C8—C9	131.8 (3)	C19—C20—C15	120.0
N3—C8—C9	105.0 (2)	C19—C20—H20	120.0
C10—C9—C8	108.3 (2)	C15—C20—H20	120.0
C10—C9—C7	128.8 (2)	C16'—C15'—C20'	120.0
C8—C9—C7	122.9 (2)	C16'—C15'—N3	111.8 (10)
N2—C10—C9	109.4 (2)	C20'—C15'—N3	125.8 (11)
N2—C10—C11	121.2 (3)	C15'—C16'—C17'	120.0
C9—C10—C11	129.4 (2)	C15'—C16'—H16'	120.0
C10—C11—H11A	109.5	C17'—C16'—H16'	120.0
C10—C11—H11B	109.5	C18'—C17'—C16'	120.0
H11A—C11—H11B	109.5	C18'—C17'—H17'	120.0
C10—C11—H11C	109.5	C16'—C17'—H17'	120.0
H11A—C11—H11C	109.5	C19'—C18'—C17'	120.0
H11B—C11—H11C	109.5	C19'—C18'—H18'	120.0
N2—C12—C13'	112.3 (9)	C17'—C18'—H18'	120.0
N2—C12—C13	107.8 (9)	C18'—C19'—C20'	120.0
N2—C12—H12A	110.1	C18'—C19'—H19'	120.0
C13'—C12—H12A	118.2	C20'—C19'—H19'	120.0
C13—C12—H12A	110.1	C19'—C20'—C15'	120.0
N2—C12—H12B	110.1	C19'—C20'—H20'	120.0
C13—C12—H12B	110.1	C15'—C20'—H20'	120.0
			
C10—N2—N3—C8	5.6 (3)	C12—N2—C10—C11	13.0 (4)
C12—N2—N3—C8	168.3 (2)	C8—C9—C10—N2	2.0 (3)
C10—N2—N3—C15'	161.9 (8)	C7—C9—C10—N2	179.5 (2)
C12—N2—N3—C15'	−35.5 (8)	C8—C9—C10—C11	−177.2 (3)
C10—N2—N3—C15	168.7 (9)	C7—C9—C10—C11	0.3 (5)
C12—N2—N3—C15	−28.7 (9)	C10—N2—C12—C13'	77.9 (7)
C7—S1—C1—C6	1.10 (18)	N3—N2—C12—C13'	−81.3 (7)
C7—S1—C1—C2	179.7 (2)	C10—N2—C12—C13	64.1 (7)
C6—C1—C2—C3	−0.8 (4)	N3—N2—C12—C13	−95.2 (7)
S1—C1—C2—C3	−179.3 (2)	N2—C12—C13—C14	−142.4 (14)
C1—C2—C3—C4	−0.1 (4)	C13'—C12—C13—C14	106 (7)
C2—C3—C4—C5	0.8 (5)	N2—C12—C13'—C14'	−108 (2)
C3—C4—C5—C6	−0.6 (5)	C13—C12—C13'—C14'	−35 (6)
C7—N1—C6—C1	0.3 (3)	C8—N3—C15—C16	−133.0 (8)
C7—N1—C6—C5	179.4 (3)	N2—N3—C15—C16	65.4 (10)
C2—C1—C6—N1	−179.8 (2)	C15'—N3—C15—C16	91 (6)
S1—C1—C6—N1	−1.1 (3)	C8—N3—C15—C20	60.6 (8)
C2—C1—C6—C5	1.0 (4)	N2—N3—C15—C20	−101.0 (10)
S1—C1—C6—C5	179.8 (2)	C15'—N3—C15—C20	−75 (6)
C4—C5—C6—N1	−179.4 (3)	C20—C15—C16—C17	0.0
C4—C5—C6—C1	−0.3 (4)	N3—C15—C16—C17	−165.3 (9)
C6—N1—C7—C9	−177.2 (2)	C15—C16—C17—C18	0.0
C6—N1—C7—S1	0.6 (3)	C16—C17—C18—C19	0.0
C1—S1—C7—N1	−1.01 (19)	C17—C18—C19—C20	0.0
C1—S1—C7—C9	176.89 (19)	C18—C19—C20—C15	0.0
N2—N3—C8—O1	176.1 (3)	C16—C15—C20—C19	0.0
C15'—N3—C8—O1	23.3 (8)	N3—C15—C20—C19	167.6 (9)
C15—N3—C8—O1	10.8 (7)	C8—N3—C15'—C16'	−121.9 (9)
N2—N3—C8—C9	−4.3 (3)	N2—N3—C15'—C16'	87.4 (7)
C15'—N3—C8—C9	−157.0 (7)	C15—N3—C15'—C16'	−71 (6)
C15—N3—C8—C9	−169.5 (7)	C8—N3—C15'—C20'	40.7 (11)
O1—C8—C9—C10	−178.9 (3)	N2—N3—C15'—C20'	−110.1 (9)
N3—C8—C9—C10	1.4 (3)	C15—N3—C15'—C20'	92 (6)
O1—C8—C9—C7	3.4 (5)	C20'—C15'—C16'—C17'	0.0
N3—C8—C9—C7	−176.2 (2)	N3—C15'—C16'—C17'	163.7 (10)
N1—C7—C9—C10	7.8 (4)	C15'—C16'—C17'—C18'	0.0
S1—C7—C9—C10	−169.9 (2)	C16'—C17'—C18'—C19'	0.0
N1—C7—C9—C8	−175.0 (2)	C17'—C18'—C19'—C20'	0.0
S1—C7—C9—C8	7.2 (3)	C18'—C19'—C20'—C15'	0.0
N3—N2—C10—C9	−4.7 (3)	C16'—C15'—C20'—C19'	0.0
C12—N2—C10—C9	−166.3 (3)	N3—C15'—C20'—C19'	−161.3 (9)
N3—N2—C10—C11	174.6 (2)		

### Hydrogen-bond geometry (Å, °)

**Table:**

*D*—H···*A*	*D*—H	H···*A*	*D*···*A*	*D*—H···*A*
C2—H2···O1^i^	0.93	2.59	3.318 (3)	135
C12—H12A···O1^ii^	0.97	2.51	3.404 (4)	152
C12—H12C···O1^ii^	0.97	2.48	3.404 (4)	159

Symmetry codes: (i) −*x*+1, −*y*+1, −*z*+1; (ii) −*x*+3/2, *y*+1/2, *z*.
